# The role of antimicrobial peptides in the evolution of endosymbiotic protein import

**DOI:** 10.1371/journal.ppat.1009466

**Published:** 2021-04-15

**Authors:** Oliver D. Caspari, Ingrid Lafontaine

**Affiliations:** UMR7141, Institut de Biologie Physico-Chimique (CNRS/Sorbonne Université), Paris, France; Tufts Univ School of Medicine, UNITED STATES

## Introduction

Antimicrobial peptides (AMPs) represent an ancient mechanism for antagonizing microbial opponents, being generated by eukaryotes, eubacteria, and archaea alike [1,2]. Given the dearth in new antibiotics, there has been increasing interest in AMPs. As our understanding has grown, a tantalizing possibility has taken shape: Might these agents of competition be at the heart of the cooperative success story that gave rise to mitochondria and chloroplasts? Striking similarities suggest that the system of protein import into these endosymbiotic organelles may derive from an interplay of AMP attack and defense [3].

## AMPs can be detoxified through import

While the term “AMP” applies to any antimicrobial based on peptide bonds, this article focuses on genetically encoded, ribosomally produced AMPs (RAMPs). Many RAMPs act by destabilizing membranes, although several also have intracellular targets [2]. The peptide nature of RAMPs allows for a general detoxification mechanism via proteolytic degradation in the bacterial cytoplasm, rendering import a viable defense strategy. First proposed in 1993 based on a screen for mutants *s*ensitive to *a*ntimicrobial *p*eptides (*sap*) in Salmonella [4], RAMP internalization was finally confirmed via immunogold labeling for *sap* homologues in non-typeable *Haemophilus influenzae* [5]. Such defense by import plays a role in extant endosymbiosis: In *Sinorhizobium meliloti*, the *BacA* transporter promotes defensive uptake of nodule-specific cysteine-rich (NCR) RAMPs expressed by the host plant [2,6]. These detoxification mechanisms have striking analogies to protein targeting to eukaryotic organelles [3], in which nuclear encoded proteins addressed to mitochondria or chloroplasts harbor highly divergent N-terminal presequences termed as “targeting peptides (TPs)” [7,8]. Translocation machineries import the targeted preprotein across 2 sets of membranes, and then the TP is cleaved by dedicated peptidases [9,10].

## HA-RAMPs and TPs are similar in structure and function

**Fig 1:**
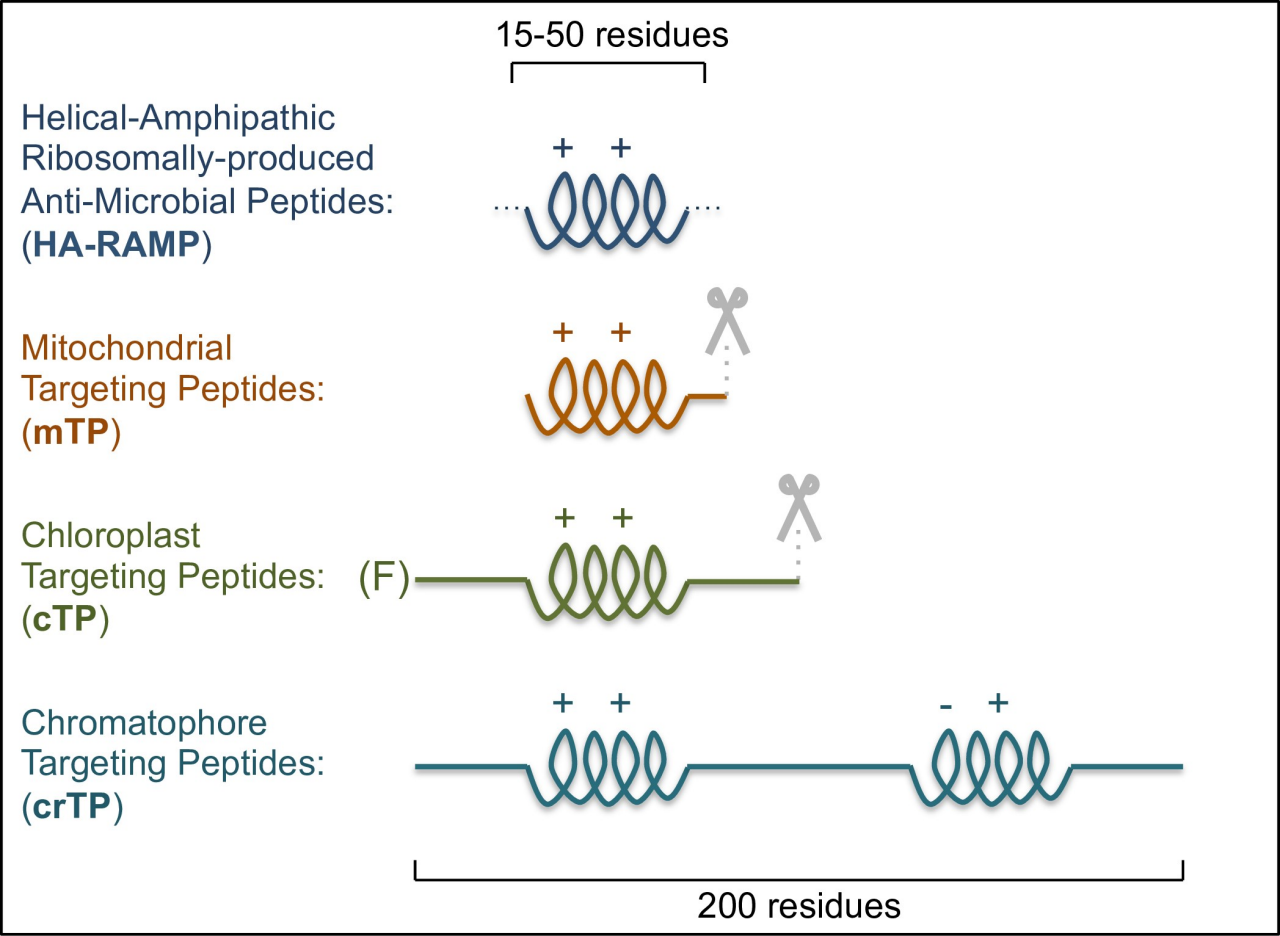
Similarities between TPs and HA-RAMPs suggest organelle import evolved from AMP detoxification. HA-RAMPs and TPs bear cationic (++), amphipathic α-helices. Further domains include cleavage sites (mTP and cTP, indicated by scissors), an uncharged N-terminus (cTP) with a leading Phenylalanine (F) in basal algal lineages, or a long carboxyl terminus (crTP) with a second predicted amphipathic helix carrying both positive and negative charges (–+).

Mitochondrial TPs (mTPs) are characterized by a cationic, amphipathic α-helix [11] (Fig 1). Chloroplast TPs (cTPs) are less structured, yet examples studied via NMR were shown to contain stretches that fold into amphipathic helices in a membrane-mimetic environment [12–14], and signatures of such stretches can be found in the majority of cTPs [15]. The main difference between mTPs and cTPs therefore lies in the presence of additional, unstructured domains (Fig 1), notably an uncharged N-terminus [7,16].

The amoeba *Paulinella* provides a further example of TPs, having acquired a photosynthetic organelle called chromatophore in a much more recent cyanobacterial endosymbiosis (ca. 60 to 100 Mya) than Archaeplastida (ca. 1 Gya) [17,18]. Many imported proteins bear putative chromatophore TPs (crTPs). These crTPs are highly similar in sequence, indicating a single common evolutionary origin [19]; crTPs are also much longer than cTPs or mTPs and may not be cleaved, all pointing to chromatophore import being very distinct [19]. Yet crTPs are imported into plant chloroplasts and contain a positively charged amphipathic helix toward the N-terminus (Fig 1).

TPs thus resemble RAMPs of helical-amphipathic structure (HA-RAMPs) [3]. A recent analysis of physicochemical properties showed these similarities to be far from superficial [15]. In a quantitative description of peptide properties, the strength of the resemblance between TPs and HA-RAMPs equals or exceeds that between different types of signal peptides known to share a common origin [15]. It may thus be of interest to revise AMP classification based on physicochemical properties, as some of the existing AMP families are defined according to criteria, such as the source organism, that offer limited information on the nature of the peptides [15].

Not only do TPs share chemical properties with HA-RAMPs, but they also interact with membranes [7] and can even have destabilizing effects [10]. Indeed, chemically synthesized TPs exhibit antimicrobial activity [15,20]. Inversely, HA-RAMPs were recently shown to target a fluorescent reporter to either mitochondria or the chloroplast in the model alga *Chlamydomonas reinhardtii* [15].

## From antagonism to endosymbiosis: Evolving inside out

The above examples of cross-functionality, rooted in a shared set of physicochemical properties, support an evolutionary link between TPs and HA-RAMPs [15]. Host attack with HA-RAMPs may have selected the bacterial organelle ancestor for defense via import. This interaction could then have been co-opted for protein import when gene transfer or genome rearrangements generated HA-RAMP–protein fusions. Having a system of protein import was crucial to enable the large-scale endosymbiotic gene transfer from the emerging organelles to the host nucleus. It was this gene transfer that generated the highly genetically integrated eukaryotic cells we know today.

Although extant import machineries of both organelles are evolutionary mosaics, containing proteins from host and bacterial ancestors alike [7,21–23], the majority of pore-forming proteins, presumably the most central and ancient factors of the import machineries, appear to have arisen within the bacterial partner during endosymbiosis. For example, the chloroplast outer envelope pore Toc75 is thought to derive from Omp85, a bacterial chaperone that aids assembly of certain outer membrane proteins recognized by carboxyl-terminal stretches ending in phenylalanine [24]. Toc75 switched polarity to accept polypeptides from the host cytoplasm rather than the bacterial periplasm [25], engendering a requirement for phenylalanine at the cTP N-terminus (Fig 1) that endures in basal algal lineages [26]. Also, the chloroplast inner envelope pore is most likely formed by Tic20/21, for which homologues have been identified in cyanobacteria [27], and the mitochondrial outer membrane pore Tom40 probably arose de novo within the proto-mitochondrion [28]. These results support the view that protein import evolved from within the endosymbiont [29], consistent with the AMP hypothesis, rather than being imposed by the host [30]. In addition, the Tim17 family, forming the inner mitochondrial pore, may also have arisen in the organelle ancestor [22,23,31].

Further support for protein import having evolved within the endosymbiont comes from proteases. After being imported, mTPs and cTPs are cleaved (Fig 1) by mitochondrial matrix processing peptidase (MPP) or chloroplast stromal processing peptidase (SPP) and further degraded by dual-targeted presequence peptidase (PreP) and organellar oligopeptidase (OOP) [9,10]. MPP, SPP, and PrepP are M16 metalloproteases, while OOP is a M3 metalloprotease. All have bacterial homologs, implying a prokaryotic origin. For example, MPP probably originated from a rickettsial putative peptidase (RPP)-like progenitor, with extant RPP being capable of cleaving mTPs [32,33]. M16 metalloproteases can even participate in protein translocation in bacteria [34].

## Descent from AMP defense accounts for TP sequence degeneracy

The high sequence degeneracy of mTPs and cTPs has led to an alternative hypothesis that TPs could easily have arisen from any given sequence. This idea is based on the observation that promiscuous extant protein import machineries accept even approximately 3% to 5% of sequences selected randomly within genomes [35,36], provided these sequences fold into cationic, amphipathic helices [37]. In this scenario, TPs would have converged with HA-RAMPs, perhaps due to a selection for membrane interaction. However, this hypothesis leaves TP proteolysis and the origin of translocases unexplained.

By contrast, the AMP hypothesis offers a comprehensive scenario for the evolution of all components of the protein import process, including TPs, translocases, and proteases [3,15]. TP sequence degeneracy may be accounted for by recruitment of a promiscuous AMP defense system for protein import, given that extant AMP importers can accept divergent RAMPs [5,6]. Once protein import was established, individual TPs may well have derived from sources other than HA-RAMP genes, as long as the emerging TP bears the physicochemical properties recognized by the import machinery.
